# Feasibility of Mindfulness-Based Stress Reduction for older adolescents and young adults with poorly controlled type 1 diabetes

**DOI:** 10.1080/21642850.2017.1415810

**Published:** 2017-12-27

**Authors:** Deborah A. Ellis, April Carcone, Richard Slatcher, Erica Sibinga

**Affiliations:** a Department of Family Medicine and Public Health Sciences, Wayne State University, Detroit, MI, USA; b Department of Psychology, Wayne State University, Detroit, MI, USA; c Center for Mind–Body Research, Johns Hopkins University, Baltimore, MD, USA

**Keywords:** Diabetes, young adults, stress

## Abstract

**Objective:**

The purpose of the study was to assess the acceptability and feasibility of Mindfulness-Based Stress Reduction (MBSR), a group-delivered intervention, to reduce stress and improve illness management among urban, older adolescents, and young adults with poorly controlled type 1 diabetes (T1D).

**Method:**

Ten older adolescents and young adults (9 females, 1 male) were recruited to participate in an MBSR group. Acceptability and feasibility were assessed based on recruitment and retention, treatment satisfaction, and changes in stress, diabetes management, and health status using a mixed-methods approach.

**Results:**

Satisfaction with MBSR was high based on both quantitative and qualitative data. Preliminary evidence was found to suggest that MBSR reduced stress and improved blood glucose levels.

**Conclusions:**

Findings from a small feasibility study suggest that MBSR could be delivered to urban older adolescents and young adults with T1D with high rates of satisfaction. Additional testing in adequately powered controlled clinical trials appears warranted.

## Introduction

The field of mind–body research has expanded exponentially in the past decade. Among the most important findings from the mind–body literature is the observation that exposure to psychological stress significantly increases vulnerability to poor health outcomes across a wide variety of physical conditions (Cohen, Janicki-Deverts, & Miller, 2007; Morey, Boggero, Scott, & Segerstrom, 2015) For persons with type 1 diabetes (T1D), stress has the potential to affect metabolic control directly through its impact on cortisol and other catecholamine hormones that affect insulin metabolism (Helgeson, Siminerio, Escobar, & Becker, 2008). It may also affect the metabolic control indirectly by interfering with the completion of diabetes management tasks such as taking insulin, testing blood glucose, or following dietary recommendations (Farrell, Hains, Davies, Smith, & Parton, 2004). Stress may also increase the psychological symptoms (i.e. depression) that negatively affect diabetes management (Helgeson, Escobar, Siminerio, & Becker, 2010; Palladino & Helgeson, 2012). Late adolescence and young adulthood may be particularly stressful developmental periods for persons with T1D due to numerous transitions into new roles and the need for increased independence, including independence with diabetes management (Arnett, 2004). Many studies have demonstrated that adolescents and young adults are more likely than children or older adults to be poorly adherent with diabetes management (Datye, Moore, Russell, & Jaser, 2015); stress may be one factor that accounts for such findings. Low-income and minority adolescents and young adults with T1D may be particularly vulnerable to the effects of stress (Hilliard et al., 2016).

It has been suggested that an individual’s perceptions of, and style of coping with stress, may have more influence upon the relationships among stress, diabetes management, and glycemic control than the amount or severity of stress that is experienced. In support of this, the use of coping styles that include wishful thinking and avoidance (not thinking about stressful events, giving up) has been found to be related to poor metabolic control among adolescents with T1D, while more active or problem-focused coping styles have been shown to be related to better metabolic control (Graue, Hanestad, Wentzel-Larsen, Oddmund, & Bru, 2004). Low-income and minority adolescents are more likely to engage in disengaged or avoidant coping than White or higher income youth with T1D (Jaser et al., 2012). In a study of 252 adolescents with T1D, Tran, Wiebe, Fortenberry, Butler, and Berg (2011) investigated the effects of ‘benefit finding’, or the ability to identify positive outcomes in the face of stress and adversity, on affective stress and diabetes management. Results suggest that benefit finding was associated with better diabetes management. Benefit finding was also found to buffer the disruptive effects of negative affective reactions to stress on diabetes management. Such findings suggest that interventions that help modify how individuals with T1D think about and/or manage stress may be important in reducing the impact of stress on health outcomes.

Because many studies have found that both stress and coping style affect diabetes health outcomes, several treatment trials have evaluated stress management interventions as a means for improving diabetes management and/or metabolic control in adolescents and adults with T1D (Boardway, Delamater, Tomakowsky, & Gutai, 1993; Hains, Davies, Parton, Totka, & Amoroso-Camarata, 2000; Snoek et al., 2001; Stenström, Göth, Carlsson, & Andersson, 2003). An additional trial conducted by Grey, Boland, Davidson, Li, and Tamborlane (2000) also delivered coping skills training and stress management interventions to adolescents with T1D, but the intervention was delivered conjointly with changes in medical management (transition to an intensive insulin regimen), so that the independent impact of the stress management intervention could not be assessed. These trials were similar in their use of group-delivered cognitive–behavioral therapy to reduce stress and improve diabetes management. Although almost all showed reductions in the level of self-reported stress, none showed significant improvements in diabetes management and only one showed significant improvements in metabolic control. Therefore, despite the large body of data suggesting the importance of improving skills to cope with stress, those stress management interventions developed to date have been ineffective in improving important health outcomes for adolescents and adults with T1D.

A new generation of behavioral therapies focus on changing the meaning that people ascribe to psychological events, rather than on trying to change or modify the events themselves (Hayes, Luoma, Bond, Masuda, & Lillis, 2006). These approaches promote non-judgmental acceptance of life events and awareness of internal responses to stress (mindfulness), rather than challenging the content of thoughts about stressful events or using problem-solving approaches to manage or reduce stressful events or their accompanying emotions. One of the best researched mindfulness treatment approaches for persons with chronic health conditions is Mindfulness-Based Stress Reduction (MBSR) (Kabat-Zinn, 2003). MBSR is an eight-week, group-delivered treatment that consists of instruction and practice in meditation, use of mindfulness techniques, and group discussions focused on the application of mindfulness practices to everyday activities. MBSR has been evaluated for the improvement of health outcomes and quality of life among adults with a variety of health conditions including chronic pain and cardiovascular conditions. It has been shown to have significant effects on a variety of important endpoints, including depression, anxiety, and disease status, in a number of randomized controlled trials (Chiesa & Serretti, 2010; Goyal et al., 2014; Merkes, 2010). However, findings from studies evaluating the effects of MBSR on diabetes-specific endpoints such as illness management or glycemic control in adults with type 2 diabetes are mixed (Hartmann et al., 2012; Rosenzweig, Reibel, Greeson, & Edman, 2007). In addition, its efficacy in persons with T1D has not been evaluated. Furthermore, few studies have been conducted with minorities in general or minority youth in particular (Black, Milam, & Sussman, 2009). A recent meta-analysis of mindfulness interventions with non-traditional populations such as African-Americans concluded that although promising, the evidence was currently insufficient to conclude that mindfulness practices were of value in improving the health of minorities (Fuchs, Lee, Roemer, & Orsillo, 2013).

The purpose of the present study was to assess the acceptability of MBSR to an urban sample of older adolescents and young adults with poorly controlled T1D and its feasibility for improving health outcomes. The ORBIT model (Czajkowski et al., 2015) provides intervention researchers with guidelines and processes for moving behavioral interventions through a variety of stages of development, from defining and refining intervention elements (Phase 1) through pilot testing in (Phase 2) and finally to adequately powered randomized clinical trials (Phase 3). The present study was consistent with Phase 1b of the ORBIT model, in which methods such as small sample case studies and qualitative data are used to establish the acceptability of the intervention to the target population and to evaluate early signals of intervention efficacy. The conduct of a Phase 1b study is considered particularly appropriate within the ORBIT model when an existing treatment, such as MBSR, is adapted to new conditions, such as a new patient population (i.e. T1D). MBSR acceptability and feasibility were assessed from several standpoints including recruitment and retention rates in the MBSR program, participant satisfaction with MBSR treatment, and preliminary evidence of efficacy for the intervention to reduce stress, improve diabetes management, and improve glycemic control.

## Materials and methods

### Participants

A convenience sample of 10 older adolescents and young adults aged 16 years, 0 months to 20 years 11 months with T1D was recruited from an endocrinology clinic located within a children’s hospital in a major Midwestern metropolitan area. The clinic served youth with T1D until the age of 21. To be study eligible, potential participants had to have been diagnosed with T1D for at least six months and to have poor metabolic control as defined by a current HbA1c ≥9% and ≤14%. Youth with an HbA1c higher than 14% were excluded from the feasibility study as potentially needing a more intensive intervention and/or diabetes-specific interventions. No exclusions were made due to co-morbid mental health problems (e.g. ADHD, depression), with the exception of conditions that might compromise data integrity or ability to participate in a group-based intervention (i.e. thought disorders/psychosis, autism, developmental delay, suicidality). Participants were excluded based on the presence of co-morbid physical health problems (e.g. cystic fibrosis) resulting in atypical diabetes management or inability to speak and read English. Participants were recruited through letters sent to their home, which were followed by phone calls to describe the study in more detail and assess interest in participating. The research was approved by the Human Investigation Committee of the university affiliated with the hospital where the adolescents were seen for medical care. All participants and/ or their legal guardian provided informed consent or assent to participate (i.e. parent provided informed consent and youth provided assent for youth under 18 years of age; participants aged 18 provided informed consent).

### Procedure

A pre–post design was used to evaluate outcomes in 10 youths who participated in an MBSR treatment group. Mixed methods were used to evaluate study outcomes. Data were collected in the two weeks immediately prior to the start of MBSR (pretest) and during the two weeks after the conclusion of treatment (post-test). All data collection visits were conducted in the home by a trained research assistant. Participants were reimbursed a total of $20 for each data collection visit.

#### MBSR intervention

MBSR is a group-delivered treatment (Kabat-Zinn, 2003) that consists of instruction and practice in meditation, use of mindfulness techniques, and group discussions focused on the application of mindfulness practices to everyday activities. MBSR intervention content is focused on the implementation of practices that enhance non-judgmental, present-focused awareness that, in turn, are thought to reduce dysregulated focusing on the past (rumination/depression) and worrying about the future (anxiety). Examples of such content include (1) practicing a variety of mindfulness meditations, including sitting meditation, walking meditation, body scan, and mindful movement/gentle yoga; (2) learning to pay attention to, observe, and describe moment-to-moment experience in a way that promotes acceptance; (3) understanding stress reactions (stress symptoms, causes, and effects) and how to create a mindful response to stressful situation; and (4) applying mindfulness to everyday activities including eating, listening, communicating, walking, and other activities. Sessions are sequential, with material presented in later sessions building on that presented in earlier ones.

In the present study, a modified version of MBSR developed for use with urban youth consisting of nine weekly sessions was utilized (Sibinga, Webb, Ghazarian, & Ellen, 2016; Sibinga et al., 2011, 2013). The MBSR program as adapted for urban youth retains the overall content, structure, and order of MBSR as used with adults (Kabat-Zinn, 1990). However, an adapted workbook for use with adolescents and young adults includes language and images appropriate for a youth population and eliminates the use of an all-day workshop by integrating this content into regular weekly group sessions. Sessions were delivered in a clinical research center at the university where the study was conducted in a large group treatment room conducive to group activities and practice of meditation and yoga activities. Participants were offered transportation to/from the group and a meal before the group. In addition, attendance at MBSR sessions was incentivized by providing a $25 gift certificate to one group attendee each week during a random drawing at the end of each MBSR session. Each session lasted approximately 90 min.

Treatment was provided by a Ph.D. level, African-American therapist who was formally trained in MBSR through attendance at a seven-day training retreat offered through the Omega MBSR Training Institute. As noted above, the therapist used a formal treatment manual to provide the MBSR treatment. Supervision was provided by weekly supervisory meetings with a local MBSR expert as well as by monthly consultations with the developers of the youth MBSR program. Treatment fidelity was monitored by a review of content checklists completed by the therapist to ensure that core content from each session was delivered, as well as by review of session audiotapes.

### Measures

#### Demographics

Participants completed the Family Information Form, an investigator-developed measure of demographic characteristics such as age, gender, race/ethnicity, family structure, living situation, employment, education, and income level.

#### Stress

The Diabetes Stress Questionnaire (Boardway et al., 1993) is a self-report instrument designed to measure day-to-day stressors encountered when managing diabetes. The instrument measures stress due to worrying about diabetes and health, peer and family interactions around diabetes, diabetes management responsibilities, and impact of symptoms such as hypo- and hyperglycemia. Sample items included ‘People finding out I have diabetes and asking me questions about it’, ‘Disagreements with family members about taking my insulin on time’, and ‘Having to live my life around a schedule of blood tests, meals and insulin’. Eight items from the original scale that were not applicable to current diabetes management practices were dropped (e.g. ‘watching brothers or sisters eat foods that I should not’) leaving a total of 45 items. In addition, items referring to stressors ‘at school’ were reworded to ‘at school or work’ since these did not apply well to older youth attending college. Participants indicated how stressful each situation was on a 4-point Likert scale (1 = Not at All, 4 = Very Much). The scale was constructed by calculating the mean of the values of the items with higher scores indicating more stress. Reliability was excellent (*α* = .96).

#### Diabetes management

Regimen adherence was measured using the Diabetes Management Scale (DMS) (Frey, Ellis, Naar-King, & Greger, 2004). The DMS is a self-report questionnaire designed to measure a broad range of diabetes management behaviors, such as insulin management, dietary management, blood glucose monitoring, and symptom response. Adolescents are asked ‘What percent of the time do you (take your insulin)?’ and answer on a 0–100% scale. Adequate reliability and validity have been reported (Schilling, Grey, & Knafl, 2002). Items are summed to obtain a total score reflecting overall management behavior. Higher scores represent better diabetes management.

#### Metabolic control

Metabolic control was calculated using hemoglobin A1c (HbA1c). Values were obtained using the Accubase A1c test kit, which is FDA approved. The test uses a capillary tube blood collection method instead of venipuncture and is therefore suitable for home-based data collection by non-phlebotomists. High-performance liquid chromatography is used to analyze the blood sample. However, HbA1c is a retrospective measure of blood glucose levels over the last 2–3 months and the intervention was only two months in length. Therefore, in order to obtain another measure of glycemic control that reflected a period of time more closely equivalent to the intervention period, mean blood glucose levels were also obtained from participants’ home blood glucose meter*.* Values for the seven-day period immediately before and immediately after MBSR were obtained. All blood glucose values were then summed and averaged.

#### Intervention satisfaction

Participant satisfaction with the MBSR intervention was measured in two ways. First, participants completed a three item questionnaire regarding their satisfaction with the intervention: (1) How helpful do you think the MBSR program was in helping you manage stress? (2) How helpful do you think the MBSR program was in helping you take care of your diabetes? and (3) If you knew another person your age with diabetes, how likely would you be to recommend the MBSR program to them? Participants responded on a 1–10 scale with 10 being highest. Second, trained research assistants completed semi-structured, face-to-face interviews designed to elicit participants’ feedback on the intervention’s appropriateness and utility. Interview questions were phrased to be open-ended, e.g. ‘Can you tell me what you thought about the MBSR program?’. Research assistants were trained to use neutral prompts, such as ‘Could you say more about that?’ to encourage participants to describe their treatment experience in their own words. Exit interviews were audio-recorded and later transcribed by a professional transcription service.

### Analytic approach

For quantitative treatment outcomes, MBSR feasibility was assessed by use of paired *t*-tests to evaluate statistical significance. Clinically significant change was also evaluated where such metrics existed for the outcome variable in question. Scatterplots were reviewed for outliers prior to analyses to ensure that a single case did not account for findings; none were observed. In addition, no outliers were identified based on screening for extreme values exceeding +/– three standard deviations from the mean (Barnett & Lewis, 1994). Qualitative data from exit interviews were analyzed using a thematic analysis approach (Aronson, 1995; Braun & Clarke, 2006). Coding was conducted using NVivo 10 (QSR International, 2014), a qualitative data analysis software package. Two coders coded the exit interviews using the following procedure. First, each transcript was independently coded using an initial list of content areas derived from the interview guide. These included helpfulness/usefulness of the intervention for stress management and diabetes management, impact on stress management and diabetes management, barriers and facilitators to participation in the intervention, and the utility of the specific content, structure, and format of intervention sessions. Coders then met to compare their coded transcripts and to discuss and resolve coding discrepancies. Next, coders used the consensus coded transcript to identify commonalities, or themes, that cut across the initial content areas. Finally, coders again coded independently, met to compare their findings, and reconciled coding discrepancies in order to achieve consensus.

## Results

The mean age of the participants was 18.6 years (*SD *= 1.2) and most (9 of 10) were female. Eight were African-American, 1 was White and I was Asian, which was representative of the demographics of the clinic where participants were recruited. Participants’ mean HbA1c was 10.6% (*SD *= 2.0%) which was well above the American Diabetes Association’s recommendation that HbA1c levels for adolescents be maintained at or below 7.5% (Silverstein et al., 2005). About a third (30%) were attending college and the remaining participants were in high school. Half (50%) reported holding part- or full-time employment and 100% reported that they still lived at home with parents or other family members. The mean length of diagnosis with diabetes was 6.2 years (*SD *= 4.8 years). Most were on intensive insulin, 20% used an insulin pump and 50% used basal-bolus injection therapy, and 30% used a regimen of 2–3 daily injections of mixed short and intermediate acting insulin. Rates of insulin pump use were consistent with those of other studies showing low rates of use in low-income and minority samples (Lin et al., 2013).

### Treatment recruitment, retention, and dose

The targeted size for the MBSR group was 10 participants. Seventy-four youths who were eligible to participate based upon age, HbA1c, and diagnosis based upon medical chart review were contacted over the phone to provide information about the study, and complete eligibility screening. Of these, two were subsequently determined not to be eligible because they resided too far from the location of the MBSR group, leaving 72 eligible participants who agreed to be re-contacted when enrollment began. For the purposes of this feasibility study, participants were systematically re-contacted until the targeted 10 were enrolled. Twenty eligible participants, or 28%, refused participation at the time of initial contact. Common reasons for lack of participation were disinterest in treatment, being too busy, or scheduling issues (e.g. attendance at college away from home, work schedule).

Of the 10 participants enrolled in the study, 3 dropped out after baseline data collection and did not participate in any MBSR treatment sessions. Those who attended any MBSR sessions did not differ from those who dropped out on age (19.5 versus 19.3 years, n.s.), gender (85% female versus 100% female, n.s.), or employment status (42% versus 50%, n.s.). For the seven who attended group, the mean number of sessions attended was six (*SD *= 2.4, range* *= 3–9). Three participants attended 3–4 sessions and four attended 6–9 sessions. MBSR treatment ‘completion’ has previously been defined as attending at least four sessions (Fjorback, Arendt, Ørnbøl, Fink, & Walach, 2011). From this perspective, 6 of 10 enrolled participants completed treatment and 6 of 7 who attended any treatment session completed treatment.

### Treatment outcomes

Table 1 shows treatment effects on diabetes-related stress, diabetes management, and glycemic control for the seven participants who completed more than a 0 dose of MBSR. From pre-treatment to post-treatment, youth reported a significant decrease in the perceptions of diabetes-related stress [*t*(6) = 1.98, *p *< .05]. In addition, there was a trend toward significant effect for MBSR to improve self-reported diabetes management [*t*(6 = –1.73 *p *< .10]. The mean seven-day blood glucose values decreased significantly from pre- to post-treatment [*t*(6)* *= 2.31, *p *< .05]. There was no statistically significant effect of MBSR on HbA1c. However, a 0.5% decrease in HbA1c is considered clinically significant, as sustained reductions in this range have been related to reductions in the rates of diabetes complications (Nathan, 2014). The mean reduction in HbA1c from pre to post was 0.7%.

**Table 1.** Treatment outcomes and treatment satisfaction among MBSR participants.

Variable	Mean (SD)	*p* value
*Health outcomes*		
Diabetes Stress Questionnaire	Pre 0.92 (0.46) Post 0.64 (0.64)	.04
Diabetes Management Scale	Pre 46.79 (10.60) Post 55.00 (12.27)	.06
Mean seven-day blood glucose (mg/dl)	Pre 301.5 (126.0) Post 229.8 (54.3)	.03
HbA1c	Pre 10.9% (2.3%) Post 10.2% (2.7%)	.23
*Satisfaction ratings*		
Was MBSR Helpful with Stress Management?	7.29 (1.89)	N/A
Would You Recommend MBSR to Someone Else with Diabetes?	8.29 (3.30)	N/A
Was MBSR Helpful with Improving Diabetes Care?	4.00 (2.77)	N/A

### Treatment satisfaction

#### Quantitative findings

In response to questions asking them to rate whether MBSR was helpful for stress management and whether they would recommend MBSR to others with diabetes, participants rated the intervention relatively highly, with mean scores ranging from 7.3 to 8.3 on a 1–10 scale (see Table 1). However, when asked if MBSR was helpful in improving diabetes care, ratings were relatively low (*M* = 4.0). This was noteworthy in light of the objective data suggesting that MBSR did in fact improve diabetes management and glycemic control. Therefore, although participants were satisfied with the intervention itself, they did not appear to link reductions in stress to improve diabetes care behaviors or health.

#### Qualitative findings: thematic analysis

Participants’ satisfaction with the MBSR program was summarized into three themes: general satisfaction, stress management in daily life, and impact on diabetes management. Consistent with the quantitative data, participants expressed high overall satisfaction with the MBSR program describing it as a ‘good program’, stating that they ‘really enjoyed it’, and reporting it was ‘beneficial in a lot of ways’. Three participants mentioned having the opportunity to meet and interact with other young adults, like them, living with diabetes to be an important part of why they liked the program.

I liked the class, meeting all the people that had diabetes.

I kind of actually wish they would’ve had more (sessions). [laughs] I like the group. It was very nice to interact with everyone and meet new people.

I kind of miss some of them.

Participants found the MBSR intervention sessions to be helpful for stress management. Specifically, participants found learning a variety of stress management techniques that they could use to solve problems in their daily lives and in stressful situations to be very helpful.

It has impacted [my stress management] by trying to think, think before doing. Say I was mad, did three breath breaks or think back and calm down before you even go ahead and go do it. Say I got mad at somebody and I calmed down before … in [my] reactions.

Sometimes taking a moment, three breaths, pause, overlook your situation, that can help you determine how you react to certain stuff. So I think that's very good.

I felt that [paying attention] was very helpful with handling stress and knowing what’s going on around you and noticing new things that you didn’t notice before. Because when I started doing that, I noticed a lot of things that I didn’t notice before and it was really nice. You know, to listen and, because the majority of the time I do it outside, like I did last night. I just got out of the car and just stood there and listened. It was peaceful.

I was never stressed but it did help me. One thing I have to admit, it did really help me a little with college because I always was nervous about failing college and it made me think positively about things.

In contrast with findings from the quantitative data, qualitative analyses suggested that some participants did see a link between the MBSR group and their diabetes management, particularly in regard to eating behavior.

The paying attention, that is very important. I think that's very important because if you're not paying attention or you’re reckless in your diabetes, that can worsen your health.

Because it helped me to, it showed me the importance of taking my medicine more, because if I wouldn’t take my medicine more, then I would be in bad condition, like health wise.

They helped how to eat mindfully and how to not eat so fast. Because you watch what you eat and make sure you don't eat too much.

Because now that I started doing the program, I thought about everything that I had to do during the visits. [laughs] and I’m like, ‘I should start doing this on a daily basis’. You know, thinking about what I’m eating and making sure I’m eating right.

## Discussion

The purpose of the present study was to obtain preliminary evidence of the acceptability of MBSR for a diverse population of 16–20-year-olds with poorly controlled T1D and the feasibility of delivering MBSR to the population. According to the ORBIT model, such studies are useful as an early stage in the testing of behavioral interventions, as lack of acceptability to the target population or inability to deliver the intervention as intended could signal the need to modify the intervention prior to moving to a fully powered clinical trial. In addition, we assessed for preliminary evidence of the intervention’s efficacy to improve treatment outcomes of importance to the health of persons with T1D, including stress, diabetes management, and health status. Older adolescents and young adults with T1D have been widely reported to be at elevated risk for poor illness management and inadequate glycemic control as compared to both younger children and older adults, as well as for poor engagement with the healthcare system (Baucom et al., 2015; Weissberg-Benchell, Wolpert, & Anderson, 2007). Emerging adulthood is a time of identity exploration, development of new social networks, and increased choices and independence (Arnett, 2000; Black et al., 2009). Therefore, while risks of poor illness management and out-of-range blood glucose are serious, youth in this age group may see these concerns as less relevant to their current life circumstances and may not be focused on improving diabetes care or motivated to receive treatment services.

Results of the present study suggest that it was feasible to deliver MBSR to older adolescents and young adults with T1D. Seventy-two percent of older adolescents and young adults who were contacted were willing to participate in the study and 70% of those who were enrolled did subsequently attend the MBSR group. On average, those who participated attended over half of the nine-session group intervention. While provision of transportation to the group as part of the study research protocol minimized one pragmatic barrier to attendance and a small incentive for attendance was offered, participants were involved in a number of activities typical of this age group (school/college attendance, work, after-school activities) and, therefore, the relatively high participation and retention rates likely reflect the value participants placed on participation rather than simply motivation to receive a gift card.

Results of the present study suggested that participants may have experienced reductions in stress after receipt of MBSR (Fjorback et al., 2011). Blood glucose levels also appeared to improve at the conclusion of the intervention. MBSR may be able to exert effects on diabetes heath status by improving illness management, improving glycemic control directly via improved cortisol regulation or both. Additional studies with larger samples are needed both to confirm MBSR’s effects on stress and diabetes-specific endpoints and to better understand how MBSR might improve glycemic control. Recent reviews (Baer, 2010; Riley & Park, 2015; Tang, Hölzel, & Posner, 2015; Witkiewitz et al., 2014) suggest that mindfulness practices may not only improve stress tolerance and emotion regulation but that they may also improve focus and attention regulation, resulting in fewer overlearned (and negative) behavioral patterns and a more intentional, active state. Such pathways warrant further explication in future studies of MBSR with youth with poorly controlled T1D, since poor self-regulation has been linked to poor diabetes management and health outcomes in this population (Berg et al., 2014; McNally, Rohan, Pendley, Delamater, & Drotar, 2010; Miller et al., 2013).

Both quantitative and qualitative satisfaction ratings suggested that the acceptability of the intervention to the target population was high. In qualitative interviews, participants found the specific skills that they were taught during MBSR to be useful in their day-to-day lives for managing a variety of day-to-day stressors and enjoyed the opportunity to connect with other young people with T1D. Findings were mixed regarding whether participants saw these improved stress management skills as contributing to improved diabetes health, since MBSR as a treatment approach does not provide disease-specific content and therefore diabetes management was not a focus of the group. Quantitative ratings of the utility of MBSR for helping participants manage their diabetes were low, despite objective data suggesting that diabetes care did in fact improve and qualitative data suggesting that participants believed MBSR treatment contributed to improved eating habits. A few participants reported that the intervention might be more useful for youth with diabetes who were experiencing a high level of stress and that they would have preferred more specific information about how to improve diabetes management skills. Future studies using MBSR for this population may benefit from the inclusion of psychoeducation that more clearly explains the relationship between stress management skills and improved diabetes care behavior or diabetes health during initial treatment sessions. It may also be beneficial to integrate more specific information regarding diabetes management into the program, although it is important to note that diabetes management education was available to study participants through the medical clinic where they received their care.

It is also noteworthy that MBSR was acceptable to a diverse, urban sample comprised largely of African-American youth given the lack of studies of mindfulness practices with minority populations (Black et al., 2009) and the higher rates of poor illness management and elevated glycemic control found in this group. There may be aspects of mindfulness practices such as MBSR that are particularly congruent with African-American cultural practices, including similarities between meditation and spirituality (Woods-Giscombé & Gaylord, 2014). The delivery of the MBSR intervention in the current study by an African-American therapist who was able to model the use of mindfulness practices may also have increased the acceptability of MBSR for African-American participants.

Limitations of the present study include the predominantly female sample, which may have affected the findings, particularly if females responded differently to a group treatment format than males. Additional limitations include the small sample size and the lack of a control group. While promising, findings that suggest MBSR may have reduced stress and improved diabetes management require replication in adequately powered studies using randomized controlled trial methods before definitive conclusions can be drawn. Due to the lack of a control group, the present study cannot assess the degree to which those generic social support processes inherent in group treatment may have accounted for some of the treatment outcomes. In addition, given the pilot nature of the study, whether or not any improvements in stress or health may have been maintained over time is unknown.

## Conclusions

Findings from the present study suggest that an urban sample of older adolescents and young adults with T1D could be recruited and retained in MBSR with high rates of satisfaction. Group-delivered mindfulness approaches such as MBSR address the needs of this age group in a number of ways, but young adults with chronic health conditions may have the additional benefit of connecting them with peers with the same health problems. Given the low base rates of many childhood chronic health conditions, many young adults lack opportunities for such contacts. Group interventions also allow for multiple youth to be provided with services by one interventionist, which may improve access to care and also reduce care costs. Additional studies are warranted to determine the efficacy of MBSR to improve health outcomes in this population.
